# Bone Marrow Derived Mesenchymal Stromal Cells Promote Vascularization and Ciliation in Airway Mucosa Tri-Culture Models *in Vitro*

**DOI:** 10.3389/fbioe.2022.872275

**Published:** 2022-06-17

**Authors:** Anja E. Luengen, Maria Cheremkhina, Julian Gonzalez-Rubio, Jan Weckauf, Caroline Kniebs, Hendrik Uebner, E. Miriam Buhl, Christian Taube, Christian G. Cornelissen, Thomas Schmitz-Rode, Stefan Jockenhoevel, Anja Lena Thiebes

**Affiliations:** ^1^ Department of Biohybrid and Medical Textiles (BioTex), AME - Institute of Applied Medical Engineering, Helmholtz Institute, RWTH Aachen University, Aachen, Germany; ^2^ Aachen-Maastricht Institute for Biobased Materials, Faculty of Science and Engineering, Maastricht University, Brightlands Chemelot Campus, Geleen, Netherlands; ^3^ Department of Pulmonary Medicine, University Medical Center Essen—Ruhrlandklinik, Essen, Germany; ^4^ Institute of Pathology, Electron Microscopy Facility, RWTH Aachen University Hospital, Aachen, Germany; ^5^ Clinic for Pneumology and Internal Intensive Care Medicine (Medical Clinic V), RWTH Aachen University Hospital, Aachen, Germany

**Keywords:** airway tissue engineering, mucociliary clearance, vascularization, air-liquid interface, mesenchymal stromal cells, 3D-lung culture, lung regeneration, primary respiratory cells

## Abstract

Patients suffering from irresectable tracheal stenosis often face limited treatment options associated with low quality of life. To date, an optimal tracheal replacement strategy does not exist. A tissue-engineered tracheal substitute promises to overcome limitations such as implant vascularization, functional mucociliary clearance and mechanical stability. In order to advance a tracheal mucosa model recently developed by our group, we examined different supporting cell types in fibrin-based tri-culture with primary human umbilical vein endothelial cells (HUVEC) and primary human respiratory epithelial cells (HRE). Bone marrow-derived mesenchymal stromal cells (BM-MSC), adipose-derived mesenchymal stromal cells (ASC) and human nasal fibroblasts (HNF) were compared regarding their ability to promote mucociliary differentiation and vascularization *in vitro*. Three-dimensional co-cultures of the supporting cell types with either HRE or HUVEC were used as controls. Mucociliary differentiation and formation of vascular-like structures were analyzed by scanning electron microscopy (SEM), periodic acid Schiff’s reaction (PAS reaction), two-photon laser scanning microscopy (TPLSM) and immunohistochemistry. Cytokine levels of vascular endothelial growth factor (VEGF), epidermal growth factor (EGF), interleukin-6 (IL6), interleukin-8 (IL8), angiopoietin 1, angiopoietin 2, fibroblast growth factor basic (FGF-b) and placenta growth factor (PIGF) in media supernatant were investigated using LEGENDplex™ bead-based immunoassay. Epithelial morphology of tri-cultures with BM-MSC most closely resembled native respiratory epithelium with respect to ciliation, mucus production as well as expression and localization of epithelial cell markers pan-cytokeratin, claudin-1, α-tubulin and mucin5AC. This was followed by tri-cultures with HNF, while ASC-supported tri-cultures lacked mucociliary differentiation. For all supporting cell types, a reduced ciliation was observed in tri-cultures compared to the corresponding co-cultures. Although formation of vascular-like structures was confirmed in all cultures, vascular networks in BM-MSC-tri-cultures were found to be more branched and extended. Concentrations of pro-angiogenic and inflammatory cytokines, in particular VEGF and angiopoietin 2, revealed to be reduced in tri-cultures compared to co-cultures. With these results, our study provides an important step towards a vascularized and ciliated tissue-engineered tracheal replacement. Additionally, our tri-culture model may in the future contribute to an improved understanding of cell-cell interactions in diseases associated with impaired mucosal function.

## Introduction

Among many diseases requiring tracheal resection, airway stenosis caused by long-term intubation represents the most common one (Mohsen et al., 2018). However, lung or thyroid cancer, tracheotomy or idiopathic reasons can also lead to potentially life-threatening tracheal obstruction. As endoscopic interventions suffer from high rates of restenosis and are therefore regarded as short-term options, resection represents the standard of care (Ferreirinha et al., 2020). Surgical treatment usually requires end-to-end anastomosis of the remaining tracheal segments. In adults, however, the procedure is only feasible if a maximum of half of the trachea has to be resected. Otherwise the strain on the remaining tissue increases excessively and the risk of restenosis rises. In children, this value is even as low as one-third of the total tracheal length (Etienne et al., 2018). Lung cancer is often diagnosed at a late stage, thus leading to technical inoperability due to extensive invasion of the trachea (Delaere, 2012).

Remaining alternative treatment options including grafts, stents or prostheses are associated with limited success. Main reasons are restenosis, immunological rejection, infection, granulation tissue formation, dislocation and material defects (Doss et al., 2007; Kucera et al., 2007; Etienne et al., 2018). Tracheal transplants are not suitable due to the lack of a direct vascular access and the need for immunosuppression in tumor patients (Delaere, 2012; Delaere and Van Raemdonck, 2016; Etienne et al., 2018). Delaere *et al.* doubted the use of synthetic tracheal prostheses, as the respiratory tract does not provide a sterile environment and bacterial contamination prevents successful prosthesis integration (Delaere and Van Raemdonck, 2016). In conclusion, Etienne *et al.* stated in 2018 that " [...] the ideal tracheal substitute is still unclear […]", leaving an unmet clinical need in the treatment of patients with advanced bronchial carcinoma and tracheal stenosis (Etienne et al., 2018).

To overcome these limitations, researchers attempted to find new therapeutic options by means of tissue engineering on the preclinical as well as on the clinical level (Soriano et al., 2021). Different scaffolds, for example decellularized tracheal tissues, hydrogels or membranes based on collagen or synthetic polymers, sometimes combined with one or more cell types, are investigated in order to create a replacement tissue resembling the native tissue as closely as possible (Dhasmana et al., 2020; Mahfouzi et al., 2021; Soriano et al., 2021). Lately, mesenchymal stromal cells (MSC) from varying origins have moved into the focus of regenerative medicine research due to their high multi-lineage differentiation capacity and their immunoregulatory properties (Han et al., 2019). The possibility to harvest MSC from autologous patient tissue in more or less minimal invasive procedures and the fact that MSC are known to secrete multiple growth factors and extracellular vesicles contribute to their high therapeutic potential (Mushahary et al., 2018; Cai et al., 2020).

Although remarkable progress has been made in the field of airway tissue engineering in the past few years, current strategies for created tracheal grafts still suffer from poor vascularization, inadequate epithelialization and insufficient mechanical properties (Dhasmana et al., 2020; Soriano et al., 2021). In terms of avoiding tissue necrosis, inflammation and infection, formation of a vascular network and a protective respiratory epithelium with functional mucociliary clearance is crucial. First attempts to re-vascularize tracheal grafts either focused on wrapping the constructs in host tissues like muscle flaps, on heterotopic pre-implantation, or on re-seeding cells on decellularized tracheal donor tissue. With increasing interest in 3D bioprinting approaches, the creation of pre-vascularized grafts *in vitro* by seeding endothelial cells in hydrogels or bio-inks gave new hope to solve the issue even though lack of mechanical stability remains as a major challenge with these scaffolds (Ott et al., 2011; Sun et al., 2021).

Focusing on the topics of vascularization and epithelialization, we have recently described the establishment of a vascularized and mucociliated *in vitro* tri-culture model based on human umbilical vein endothelial cells (HUVEC), human nasal fibroblasts (HNF) and human nasal epithelial cells (HNEC) in fibrin gel (Kreimendahl et al., 2019). Fibrin-based hydrogels offer great potential as they can be obtained autologously, are known to promote vascularization and can be modified to be used as injectables or bio-ink (Cornelissen et al., 2012; Park and Woo, 2018; Kniebs et al., 2020). In order to investigate beneficial effects of MSC, we now evaluated the use of adipose-derived mesenchymal stromal cells (ASC) or bone marrow-derived mesenchymal stromal cells (BM-MSC) as supporting cell type in comparison to HNF in the model. Since differences in cilia-related gene expression between nasal and tracheal epithelial cells are reported in the literature, we furthermore replaced the HNEC by human respiratory epithelial cells derived from bronchial biopsies of healthy donors (HRE) to avoid variations in cell behavior caused by location (Mihaylova et al., 2018; Imkamp et al., 2019).

**FIGURE 1:**
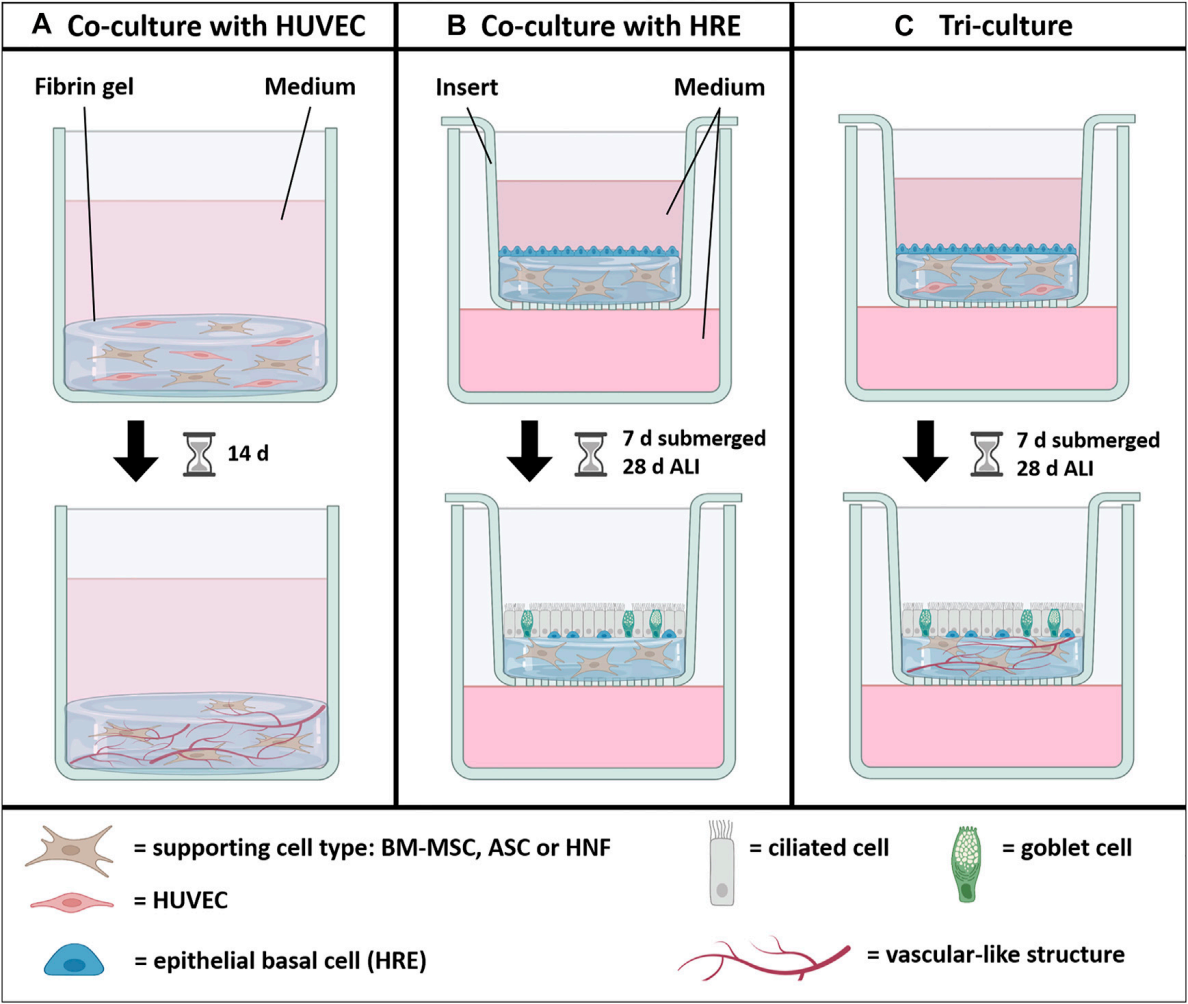
Graphical abstract. One of the supporting cell types BM-MSC, ASC or HNF was combined with either HUVEC **(A)**, HRE **(B)** or both **(C)** to investigate the influence on formation of vascular-like structures mediated by HUVEC and on mucociliary differentiation of HRE. HUVEC and the supporting cell types were seeded in fibrin gel while HRE were seeded on top. All cultures with HRE were differentiated at air-liquid interface (ALI) conditions for 4 weeks after a submerged proliferation period for 1 week. Co-cultures with HUVEC were cultured for 2 weeks. Figure created with BioRender.com.

In this study, we compared three different supporting cell types, BM-MSC, ASC and HNF, with respect to their influence on vascularization and mucociliary differentiation in a tri-culture model with HUVEC and HRE based on fibrin gel. Co-cultures containing one of the supporting cell types and either HRE or HUVEC were used as controls (Figure 1). Tri-cultures and co-cultures with HRE were established in cell culture inserts, proliferated under submerged condition and differentiated for 4 weeks under air-liquid interface (ALI) conditions. All cultures without HRE were cultured submerged for 14 days. After cultivation, epithelial differentiation, formation of vascular-like structures as well as cytokine production were analyzed.

## Materials and Methods

### Isolation and Expansion

Cell isolation procedures followed recently described protocols (Menzel et al., 2017; Kreimendahl et al., 2019; Kniebs et al., 2020; Kniebs et al., 2021; Schuhenn et al., 2022). For information on ethical approval and relevant guidelines, please refer to the declarations section.

Briefly, HRE were obtained from lung transplant donors post mortem at the University Medical Center Essen, Germany. Selection criteria for donors are listed in the Eurotransplant guidelines. Cells were isolated and cultivated following a well-established, verified and commonly used method (Mertens et al., 2017). They were expanded in keratinocyte-SF medium (KSFM, Gibco) supplemented with human EGF (Gibco, 2.5 ng/ml), bovine pituitary extract (BPE, Gibco, 25 μg/ml), isoproterenol (Sigma-Aldrich, 1 μM) and a mixture of Penicillin, Streptomycin, Ciprofloxacin and Amphotericin B (PanBiotech, 2.5 μg/ml). Afterwards, cells were frozen in liquid nitrogen using a mixture of 90% KSFM, 10% dimethyl sulfoxide (DMSO, Sigma) and BPE (0.3 mg/ml) until further use. To verify cell identities of isolated HRE, they are screened regularly by immunohistochemical staining for basal cell marker P40/chromogranin A. Furthermore, HRE were verified by their ability to differentiate in monoculture under ALI conditions into ciliated cells and mucus producing goblet cells (see Supplementary Figure S1). HUVEC were isolated from human umbilical cords provided by the Clinic for Gynecology and Obstetrics (RWTH Aachen University Hospital) and expanded in endothelial cell growth medium 2 (EGM2, Promocell) up to passage 3.

BM-MSC were isolated from femoral heads supplied by the Clinic for Orthopedic, Trauma and Reconstructive Surgery (RWTH Aachen University Hospital) and cultured in Mesenpan medium (PAN-Biotech) supplemented with 2% FCS (Gibco) and 1% antibiotic/antimycotic solution (ABM, Gibco) up to passage 4.

ASC were isolated from abdominal fat tissue obtained from the Clinic for Plastic, Hand and Burns Surgery (RWTH Aachen University Hospital) and expanded in supplemented Mesenpan medium up to passage 3.

HNF were isolated from nasal conchae received from the Clinic for Otorhinolaryngology (RWTH Aachen University Hospital) and cultured in Dulbecco`s Modified Eagle Medium (DMEM, Gibco) supplemented with 10% FCS and 1% ABM up to passage 3.

Cell identities of HUVEC, BM-MSC, ASC and HNF were verified in previous studies using the same isolation protocols (Weinandy et al., 2014; Kreimendahl et al., 2019; Kniebs et al., 2020; Thiebes et al., 2021). Additionally, isolation procedures for MSC are regularly verified by flow cytometry according to the minimal criteria for the classification of MSC given by Dominici et al. (2006): ASC and BM-MSC gave positive signals for CD90, CD73 and CD105 with ≥97.2%. The markers CD45, CD34, CD11b, CD79α and HLA-DR surface molecules were found to be negative (<1.3%).

### Molding of Fibrin Gels and Cell Seeding

Experiments were conducted with HRE in passage 1, with HUVEC, ASC and HNF in passage 4 and with BM-MSC in passage 5. BM-MSC, ASC and HNF were tested using three different donors per cell type while for HUVEC and HRE, one donor was tested per cell type leading to three repetitions per culture and supporting cell type. As high cell numbers are needed for three-dimensional cell culture and the amount of primary cells that can be isolated from fresh tissue is limited, only the supporting cell types were tested with regard to donor variation in order to evaluate their influence on the differentiation. The HUVEC donor and the HRE donor were kept constant to avoid a donor-related impact on the results not originating from the supporting cell types. Especially with regard to HRE, donor variability can be high and availability of cells derived from healthy donors is limited. In tri-culture experiments, HUVEC were mixed with either BM-MSC, ASC or HNF in a fibrin gel before HRE were seeded on top of the gel. In co-culture experiments, either HUVEC were combined with one supporting cell type (BM-MSC, ASC or HNF) in a fibrin gel or HRE were seeded on top of a gel containing one of the supporting cell types. Additionally, a co-culture of HUVEC and HRE as well as monocultures of HUVEC and HRE were prepared as controls. Fibrin gels were molded according to previously published protocols (Kniebs et al., 2020; Kreimendahl et al., 2021). Briefly, HUVEC and/or one of the supporting cell types were seeded with a final concentration of $3\cdot 10^{6}$ cells/mL each in a fibrin gel containing 5 mg/ml fibrinogen (VWR), 3 U/mL thrombin (Sigma-Aldrich) and 3.75 mM CaCl_2_. Gels were either molded in 24-well plates (VWR) for co-cultures with HUVEC and monocultures (350 µL) or in Transwell^®^ Inserts (PET membrane, 0.4 µm pore size, Corning^®^) combined with ThinCert™Plates (Greiner Bio One) when used for ALI (206 µl). Polymerization of the gels was achieved by incubation at room temperature for 20 min followed by incubation at 37°C and 5% CO_2_ in a humidified atmosphere for another 20 min. In case of co-cultures with HRE and tri-cultures, HRE were then seeded on top of the gels with a final density of 80,000 cells/cm^2^. For the HRE monoculture, cells were seeded onto Transwell inserts coated with collagen IV from human placenta (12 μg/cm^2^, Sigma-Aldrich). Detailed media composition was described previously (Kreimendahl et al., 2019; Luengen et al., 2020). In short, cultures with HRE except the monoculture were proliferated under submerged conditions in a 1:1-mixture of EGM2 and airway epithelial cell growth medium (AECGM, Promocell) for 1 week before switching to ALI-conditions in a 1:1-mixture (EMM) of EGM2 and MucilAir medium (Epithelix) for 4 weeks. HRE monoculture was performed using AECGM in the proliferation phase and a modified version of AECGM in the differentiation phase (mAir). Co-cultures with HUVEC as well as monocultures of BM-MSC, ASC and HNF were cultured in EMM medium for 2 weeks. In all cultures, medium was exchanged every 2–3 days. All media were supplemented with 0.05%–0.1% Gentamicin (Rotexmedica) and 0.16% tranexamic acid (Carinopharm) to avoid gel degradation.

### Evaluation of Epithelial Cell Differentiation and Vascularization

Cell differentiation was analyzed by scanning electron microscopy (SEM), two-photon scanning electron microscopy (TPLSM) and histologic and immunohistochemical stainings. To evaluate ciliation of HRE after 4 weeks of ALI, SEM analysis was conducted by the facility for electron microscopy of the medical faculty of RWTH Aachen University. After fixation in 3% glutaraldehyde (Agar scientific), samples were rinsed with 0.1 M sodium phosphate buffer (Merck) followed by dehydration in an ascending ethanol series. Subsequent to critical point drying in liquid CO_2_, samples were coated with a 10 nm gold/palladium layer (Sputter Coater EM SCD500, Leica). SEM was carried out in a high vacuum environment at 10 kV acceleration voltage with an environmental scanning electron microscope (ESEM XL30 FEG, FEI).

Vascularization of the samples was analyzed using TPLSM after previously established protocols (Kreimendahl et al., 2019; Kniebs et al., 2020). After fixation in ice-cold methanol, samples were first stained with mouse anti-CD31 (PECAM-1, 1:100; Sigma-Aldrich) and Alexa Fluor^®^ 594 goat anti-mouse IgG (1:400, Invitrogen). A selection of tri-culture samples was further stained sequentially using mouse anti-vimentin (1:100; Sigma-Aldrich), Alexa Fluor^®^ 488 goat anti-mouse IgG (1:400, Invitrogen) and DAPI (Molecular Probes). The TPLSM was performed using a two-photon laser scanning microscope (Olympus FluoView 1000 MPE) with a ×25 water-objective NA 1.05 (Olympus Optical) and a MaiTai Deep-See Titan-Sapphire-laser. FluoView FV 4.2 Software was used for the visualization while images were processed and evaluated with Imaris 9.6.0 software (Bitplane Inc. South Winsor). Vascularization was quantified by evaluating the average volume, surface area, length and number of branching points of vascular-like structures. Three images per gel were taken for the statistical result evaluation.

For histologic and immunohistochemical evaluation, samples were embedded in 2% agarose, fixed in Carnoy’s solution and dehydrated in an ascending ethanol series followed by incubation in xylene and embedding in paraffin. Native human nasal concha tissue was fixed in 4% paraformaldehyde solution before dehydration and paraffin embedding as described above. Afterwards, 5 µm-thick deparaffinized sections were stained by periodic acid Schiff’s reaction (PAS reaction) to analyze epithelial cell differentiation using a bright field light microscope. Briefly, deparaffinized samples were first hydrolyzed in 1% w/v periodic acid solution before staining with Schiff’s reagent (both Merck). After a washing step in 35°C warm tap water, cell nuclei were counterstained with Mayer’s hematoxylin (Sigma-Aldrich). Immunohistochemical analysis was also performed on Carnoy-fixed sections after a previously described protocol (Luengen et al., 2020). After deparaffinization, samples were blocked with 5% normal goat serum (NGS, Dako). Following incubation in primary antibody solutions overnight at 4°C and three washing steps, sections were incubated protected from light in secondary antibody solutions for 1 h at 37°C. Primary antibodies included rabbit anti-*pan*-cytokeratin (1:200, *Acris*), mouse anti-mucin-5AC (1:800, *Acris*), rabbit anti-claudin-1 (1:800, Biorbyt), mouse anti-acetylated tubulin (1:800, Sigma-Aldrich) and mouse anti-CD31 (PECAM-1, 1:100; Sigma-Aldrich). Fluorescently labelled secondary antibodies Alexa Fluor 488 goat anti-rabbit IgG and Alexa Fluor 594 goat anti-mouse IgG (both Invitrogen) were used for detection. All antibodies were diluted in PBS containing 1% w/v bovine serum albumin (BSA, Sigma-Aldrich) and 0.1% w/v sodium azide (Sigma-Aldrich). Counterstaining of cell nuclei was carried out using DAPI. For observation, an inverted fluorescence microscope (Axio Observer. Z1, Carl Zeiss) was used.

### Cytokine Production

To evaluate differences in signaling patterns between cultures, a LEGENDPlex™ bead-based immunoassay (BioLegend) for eight human cytokines including vascular endothelial growth factor (VEGF), epidermal growth factor (EGF), interleukin-6 (IL6), interleukin-8 (IL8), angiopoietin 1, angiopoietin 2, fibroblast growth factor basic (FGF-b), placenta growth factor (PIGF), interleukin-33 (IL33), thymic stromal lymphopoietin (TSLP) and transforming growth factor-beta1 (TGF-β1) was performed on media supernatant at the end of the ALI culture following the manufacturer’s instructions. Results were evaluated according to the manufacturers’ instructions.

### Ciliation Quantification

To compare the ciliation amount between different cultures, ten independent and in terms of mucociliary differentiation well educated observers classified SEM images blinded for the condition as previously described (Luengen et al., 2020). The scoring system provided in Table 1 was used for classification of the proportion of the ciliated area in relation to the total surface area in one general overview image of the sample. Afterwards, mean values per image and for each condition were calculated.

**TABLE 1:** Ciliation scoring system.

Score	Ciliation
0	none
1	low
2	medium
3	strong
4	very strong

### Statistical Analysis

Statistical analysis was performed using GraphPad Prism software 9.2.0. For vascularization and ciliation quantification, statistics were performed by one-way analysis of variance (ANOVA) with Tukey’s post-hoc test. Standard deviation of ciliation scores was determined by Gaussian error propagation. Cytokine production results were analyzed by two-way ANOVA with Tukey’s post-hoc test. A *p*-value of *p* < 0.05 was considered as statistically significant.

## Results

To engineer a vascularized and mucociliated respiratory mucosa based on MSC *in vitro*, tri-cultures with HRE, HUVEC and one of three different supporting cell types BM-MSC, ASC or HNF were created in fibrin gel. Furthermore, vascularization and mucociliary differentiation were tested in co-culture models of HRE or HUVEC combined with one of each of the three supporting cell types. Tri-cultures as well as co-cultures with HRE were cultured under submerged conditions for 1 week before switching to ALI conditions for 4 weeks. Co-cultures with HUVEC were vascularized for 2 weeks. At the end of the culture period, mucociliary differentiation of HRE on top of the gels and formation of vascular-like structures in the gels were analyzed using histological and immunohistochemical methods as well as SEM and TPLSM. Differences in cell communication *via* cytokine secretion were evaluated using LEGENDPlex immunoassay.

### Cell Morphology

**FIGURE 2:**
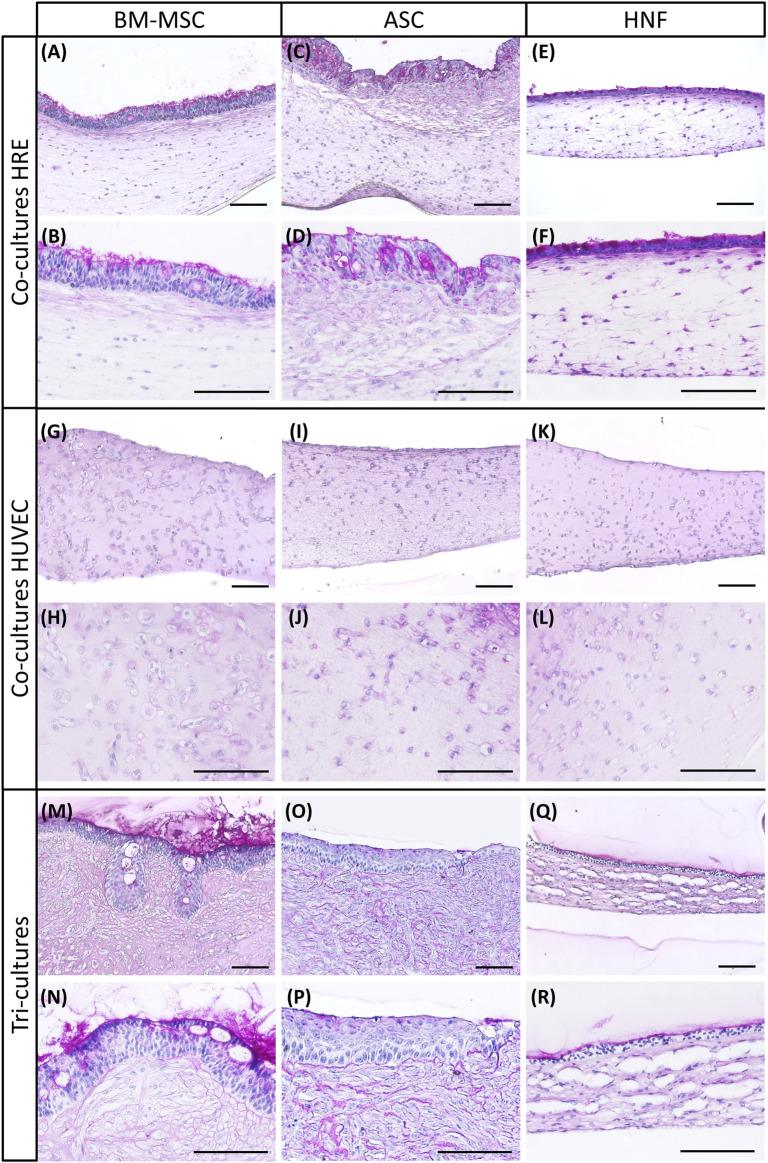
PAS reaction of co-cultures with HRE, co-cultures with HUVEC and tri-cultures. **(A–F)** *:* PAS reaction revealed presence of glycogens, mucopolysaccharides and glycoproteins in co-cultures of HRE with different supporting cell types: BM-MSC **(A,B)**, ASC **(C,D**) or HNF **(E,F)** **(G–L)**: PAS reaction showed overall morphology of fibrin gels containing HUVEC and varying supporting cell types in co-culture: BM-MSC **(G,H)**, ASC **(I,J)** or HNF **(K,L)** **(M–R)**: PAS reaction indicated near native morphology of the respiratory epithelium in tri-cultures with BM-MSC **(M,N)** while using ASC **(O,P)** or HNF **(Q,R)** led to cell multilayers with reduced mucociliary differentiation. Representative pictures of two different magnifications are shown. Scale bar: 100 µm.

Cell morphology, presence of goblet cells and mucus production in co- and tri-cultures were analyzed using PAS reaction (Figure 2). All cultures were established with three different supporting cell donors, variability between donors was found to be low. HRE-co-cultures and tri-cultures with BM-MSC revealed a characteristic and pseudostratified epithelial layer. Mucus-secreting goblet cells and basal cell morphology resembled the native respiratory epithelium (Figures 2A,B, M, N). Both HRE-co-cultures and tri-cultures with ASC showed a multilayered morphology. Mucus production confirmed in the HRE-co-cultures with ASC was mostly absent in the corresponding tri-cultures (Figures 2C,D, O, P). In addition, co-cultures with HRE and ASC lacked the clear morphology of a basal cell layer in terms of conventional histology which was observed in cultures with BM-MSC or in ASC-tri-cultures. Formation of mucus was reduced in tri-cultures with HNF in comparison to the corresponding co-cultures with HRE. However, both had a thin multilayered and rather non-pseudostratified epithelium (Figures 2E,F, Q, R). Co-cultures with HUVEC and different supporting cell types exhibited a comparable morphology (Figure 2G–L). PAS reaction images of all donors per condition as well as of native human nasal concha tissue as positive control, a co-culture of HRE and HUVEC as well as of a monoculture of HRE on inserts are shown in the supplementary (see Supplementary Figures S1–S4).

### Mucociliary Differentiation

**FIGURE 3:**
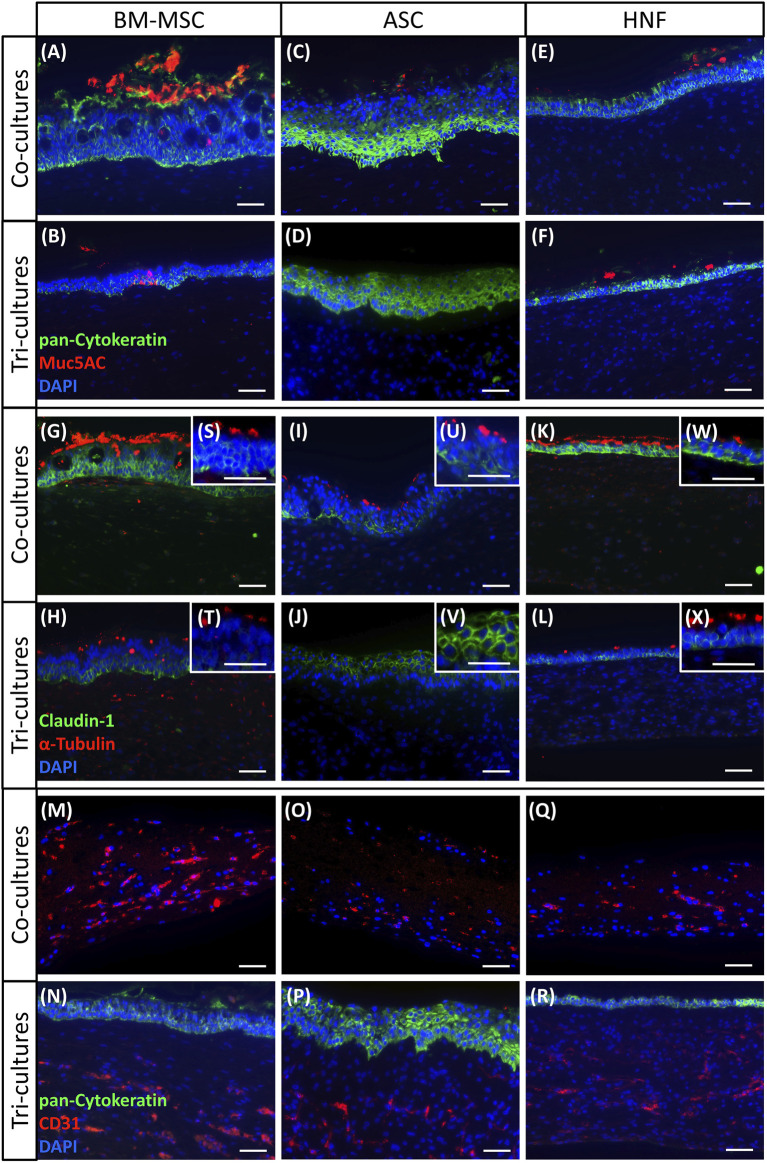
Immunohistochemical staining of co-cultures with HRE, co-cultures with HUVEC and tri-cultures. **(A–F)**: pan-cytokeratin (green) was used to detect epithelial keratins in all cultures while mucin5AC (red) showed presence of mucus and goblet cells in all cultures containing HRE with BM-MSC **(A,B)** and HNF **(E,F)** as well as in HRE-co-cultures with ASC **(C)** but not in tri-cultures with ASC **(D)** **(G–L)**: claudin-1 (green) indicated tight junction formation in all HRE-cultures, and α-tubulin (red) could visualize cilia in all HRE-cultures with BM-MSC **(G,H,S,T)** and HNF **(K,L,W,X)** as well as in HRE-co-cultures with ASC **(L,U)** but not in tri-cultures with ASC **(J,V)** **(M,O,Q)**: CD31 staining confirmed presence of endothelial cells in co-cultures of HUVEC with BM-MSC **(M)**, ASC **(O)** or HNF **(Q)** **(N,P,R)**: pan-cytokeratin stained the epithelial cell layer on top of the fibrin gel while CD31 was used to detect endothelial cells and vascular-like structures in tri-cultures containing one of the supporting cell types: BM-MSC **(N)**, ASC **(P)** or HNF **(R)**; DAPI (blue) was used to counterstain cell nuclei. Representative pictures are shown. Scale bar: 50 µm.

To confirm mucociliary differentiation and formation of vascular-like structures, immunohistochemical stainings of endothelial cell marker CD31 and of characteristic epithelial cell markers pan-cytokeratin, mucin5AC, claudin-1 and α-tubulin were performed (Figure 3). Pan-cytokeratin acts as marker for the epithelial cytoskeleton, mucin5AC reveals mucus production, claudin-1 indicates barrier formation and α-tubulin marks cilia while CD31 confirms presence of endothelial cells (Kreimendahl et al., 2019). In all cultures with HRE containing BM-MSC (Figures 3A,B,G,H, M, N, S, T), staining for the four epithelial cell markers and CD31 was positive verifying the epithelial origin of the cells as well as the presence of cilia, mucus, tight junctions and vascular-like structures comparable to native human nasal tissue as shown in the supplementary (see Supplementary Figure S5). In BM-MSC-tri-cultures, claudin-1 and pan-cytokeratin expression was additionally found to be localized in the basal cell layer only as was also observed in the native control. In co-cultures with BM-MSC, these markers were detected throughout the whole epithelial cell layer. Some unexpected α-tubulin staining was observed in BM-MSC inside gels of BM-MSC tri-cultures and co-cultures with HRE (Figures 3G,H) (Zhou et al., 2017). While all HRE-cultures containing ASC were found to be positive for pan-cytokeratin and claudin-1, cilia and mucus were only detected in co-cultures with HRE (Figures 3C,D,I,J, P, U, V). ASC-cultures containing HUVEC were stained positively for CD31 (Figures 3O, P). Moreover, claudin-1 and pan-cytokeratin expression was detected in the whole epithelial cell layer lacking the localization of these cell markers in the basal cell layer found in the native control (see Supplementary Figure S5). Although the epithelial origin of the cells could be shown by the stainings, HRE in tri-cultures with ASC failed to develop a mucociliary phenotype (Figures 3D,J,V). Cultures containing HNF were again positive for all analyzed markers although epithelial cell layers were again found to be thinner than in cultures with MSC (Figures 3E,F,K, L, Q, R, W, X). Furthermore, the characteristic distribution pattern of claudin-1 and pan-cytokeratin was also absent in all HNF-cultures which instead show expression throughout the whole epithelium. Regarding CD31 staining in co-cultures of HUVEC with supporting cell types, expression was found to be reduced in cultures with ASC and HNF in comparison with BM-MSC (Figure 3M, O, Q). Controls with native tissue, HRE and HUVEC in monoculture as well as in co-culture with each other are depicted in the supplementary (see Supplementary Figure S5).

**FIGURE 4:**
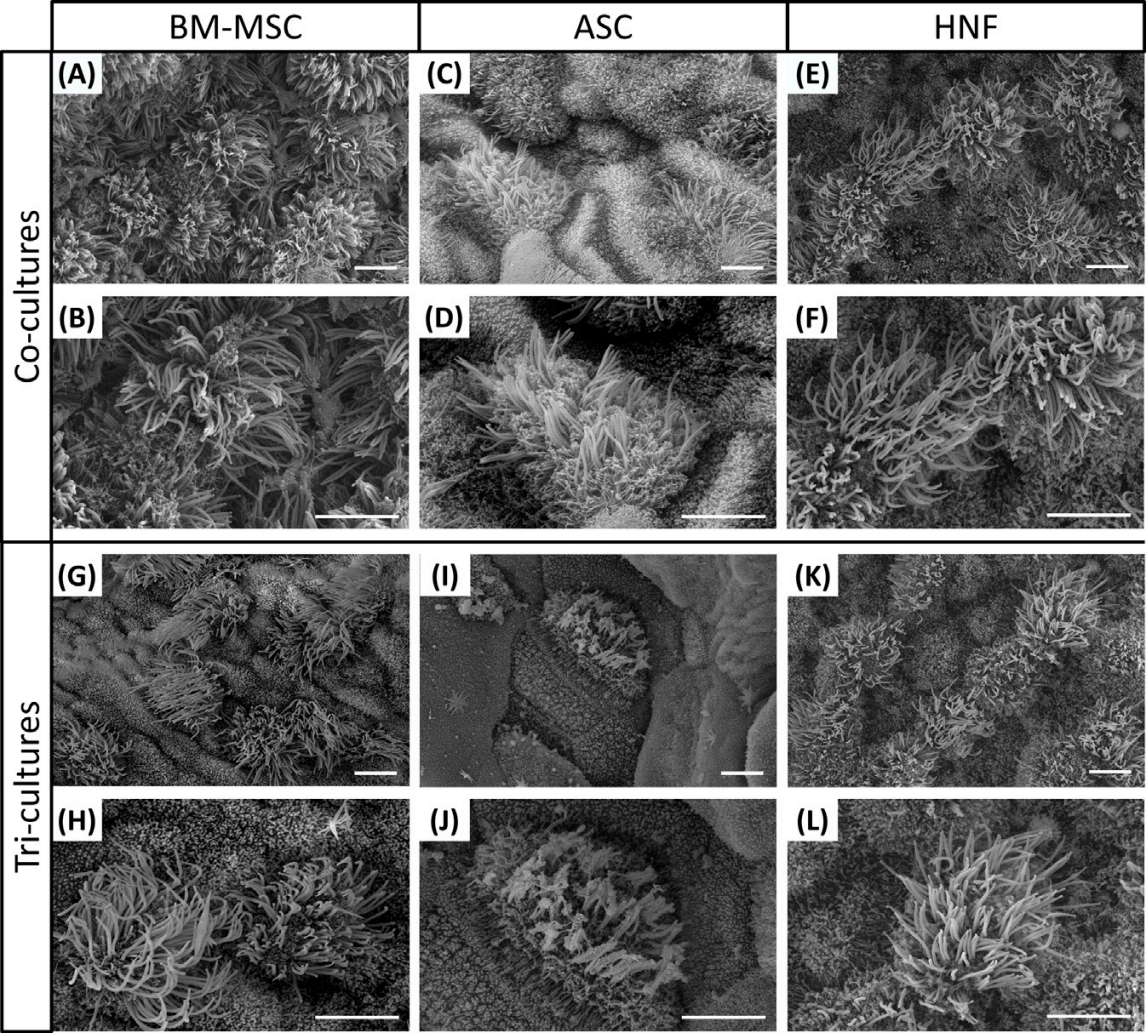
Ciliation on co-cultures with HRE and on tri-cultures after 4 weeks of ALI. **(A–L)**: SEM confirmed cilia formation on all co-cultures with HRE and on all tri-cultures; Representative images of two different magnifications are shown. Scale bar: 5 µm.

Cilia formation was additionally analyzed by SEM and could be confirmed for all cultures (Figure 4). In comparison with the HRE-co-cultures (Figures 4A,B), BM-MSC-containing tri-cultures (Figures 4G,H) were found to be slightly less ciliated. The same effect could be observed for cultures with ASC (Figures 4C,D,I,J), which additionally exhibited a lower level of ciliation in total than the cultures with BM-MSC. Cilia formation in cultures with HNF seemed to be comparable between co- and tri-cultures (Figures 4E,F,K, L).

**FIGURE 5:**
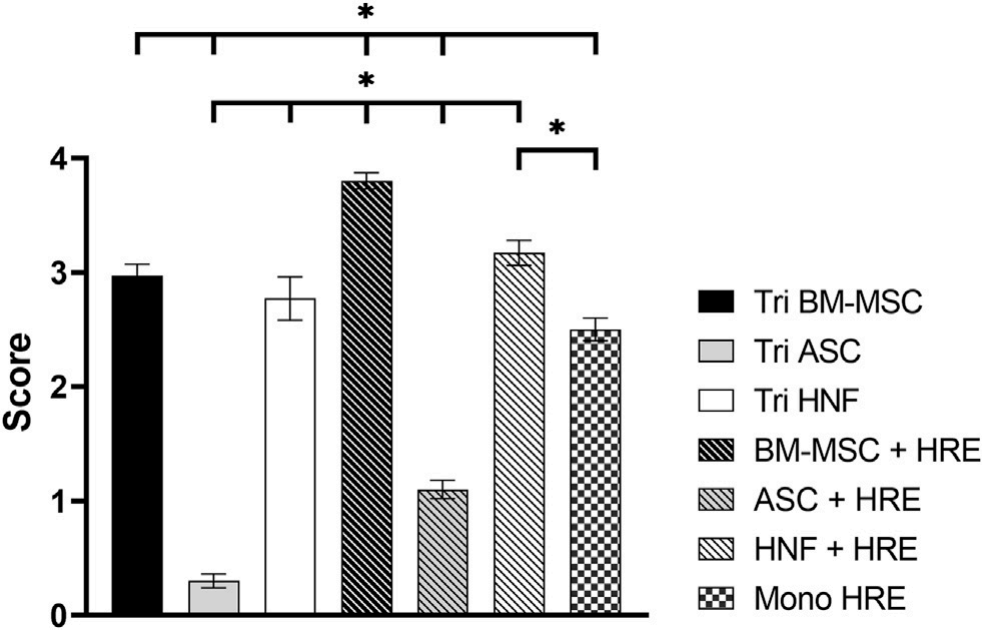
Ciliation scoring of co-cultures with HRE, tri-cultures and HRE in monoculture. Scoring results revealed statistically significant differences between most groups except tri-cultures containing BM-MSC and co-culture or tri-culture with HNF. Additionally, the HRE monoculture was found to be non-significantly different from the HNF-triculture. Results are expressed as mean ± SD following Gaussian error propagation. Statistically significant differences (*p* < 0.05) are indicated by “*”.

Estimation of the differences in ciliation amounts between different culture conditions by independent observers based on SEM images revealed significant differences between almost all groups (Figure 5): only the tri-culture with BM-MSC (mean score 2.97 ± 0.10) was found to be non-significantly different from the HNF-co-culture (mean score 3.17 ± 0.11) and HNF-tri-culture (mean score 2.77 ± 0.19). The latter was additionally observed to be non-significantly different from the monoculture of HRE (mean score 2.5 ± 0.10) that was evaluated as control group. The highest score was estimated for the co-culture with BM-MSC (mean score 3.80 ± 0.07) while the lowest scores were observed for the co-culture (mean score 1.10 ± 0.08) and the tri-culture (mean score 0.30 ± 0.06) with ASC.

### Vascularization

**FIGURE 6:**
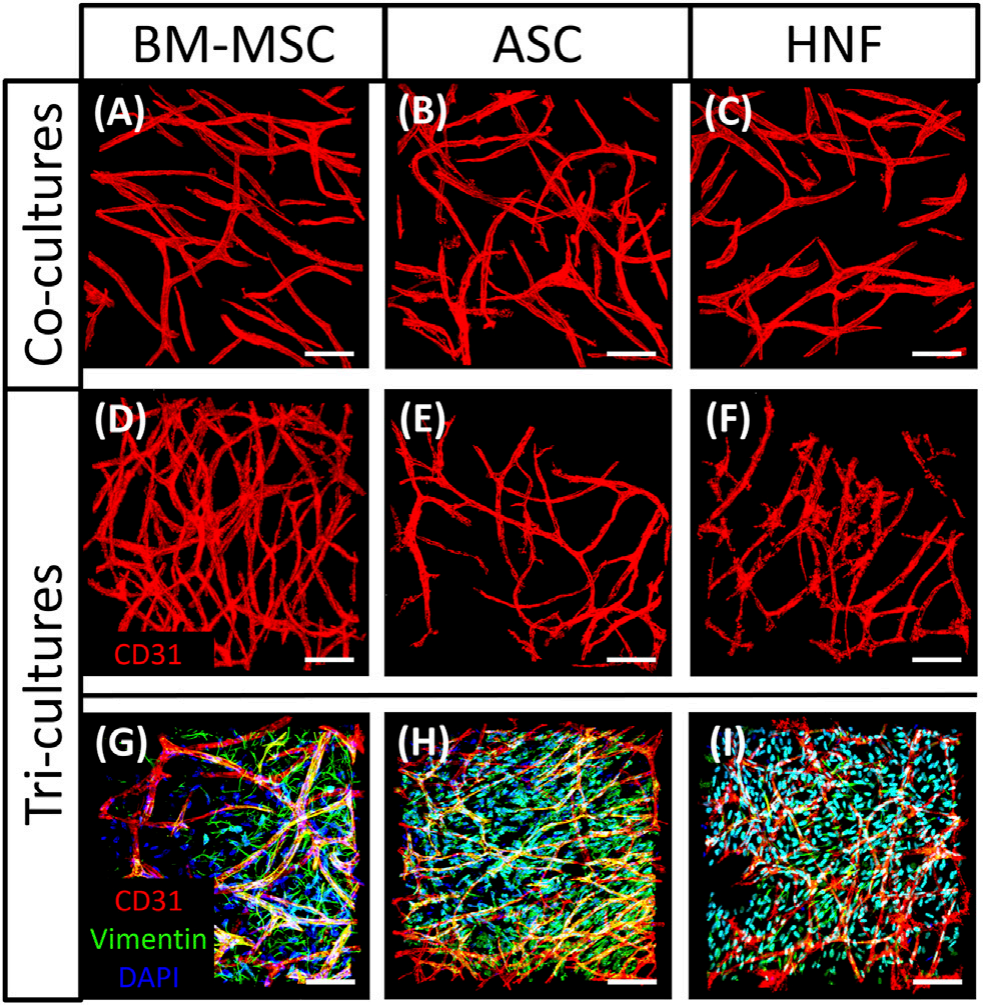
TPLSM images of co-cultures with HUVEC and tri-cultures. **(A–C)**: CD31-staining showed comparable vascularization in co-cultures of HUVEC with BM-MSC **(A)**, ASC **(B)** or HNF **(C)** **(D–F)**: larger networks of vascular-like structures were found in tri-cultures with BM-MSC **(D)** in comparison with ASC **(E)** and HNF **(F)** **(G–I)**: vimentin-staining showed distribution of supporting cell types in a selection of tri-culture-samples with BM-MSC **(G)**, ASC **(H)** or HNF **(I)**. Scale bar: 50 µm.

**FIGURE 7:**
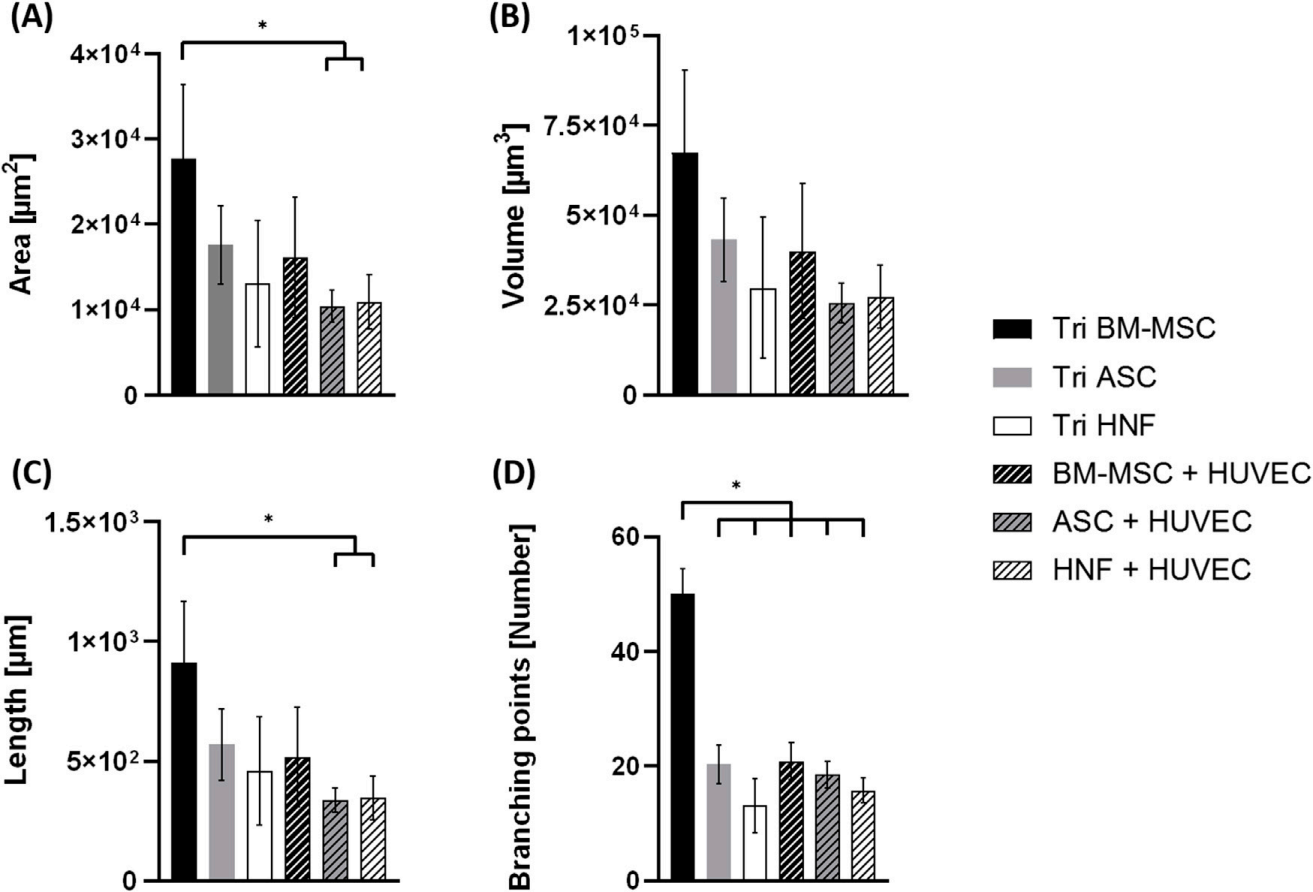
Statistical evaluation of vascularization in co-cultures with HUVEC and tri-cultures. **(A)**. area of vascular-like structures **(B)**. structure volume **(C)**. structure length **(D)**. number of branching points. Statistical significance (*p* < 0.05) is indicated by “*”.

The influence of different supporting cell types in co-cultures with HUVEC and in tri-cultures on formation of vascular-like structures was evaluated using TPLSM (Figure 6), followed by statistical analysis (Figure 7). In all cultures, elongated and branched vessel-like structures could be observed after staining with anti-CD31-antibody (Figure 6). However, tri-cultures with BM-MSC seemed to develop a larger number of thin structures that in addition appeared to be longer and more homogeneously outspread through the gel than the structures observed in other cultures (Figure 6D). While vascularization results were comparable in all co-cultures with HUVEC and in the tri-culture with HNF (Figures 6A–C,F), it seemed to be reduced in tri-cultures with ASC compared to the corresponding co-culture (Figure 6E). A selection of tri-culture samples was further stained with anti-vimentin-antibody to visualize the supporting cell type in the gel (Figures 6G–I). BM-MSC were found to be elongated and more concentrated on one side of the gel (Figure 6G) whereas ASC and HNF seemed to be smaller, not as spindle-shaped and to be more evenly distributed in the gel (Figures 6H,I). However, the observed cell distribution is caused by method-related effects of the TPLSM due to an uneven sample surface and thus not meaningful.

Statistical analysis of the TPLSM images showed significantly increased structure length and area for tri-cultures with BM-MSC in comparison to co-cultures with ASC or HNF (Figures 7A,C). In addition, the number of branching points was significantly higher in these tri-cultures in comparison to all other groups (Figure 7D). Differences observed in the volume of vessel-like structures were not found to be statistically significant (Figure *7*B). With regard to functionality of formed vascular-like structures, we could prove lumen formation independent on the supporting cell type, the corresponding images are shown in the supplementary (see Supplementary Figure S6).

### Cytokine Production

**FIGURE 8:**
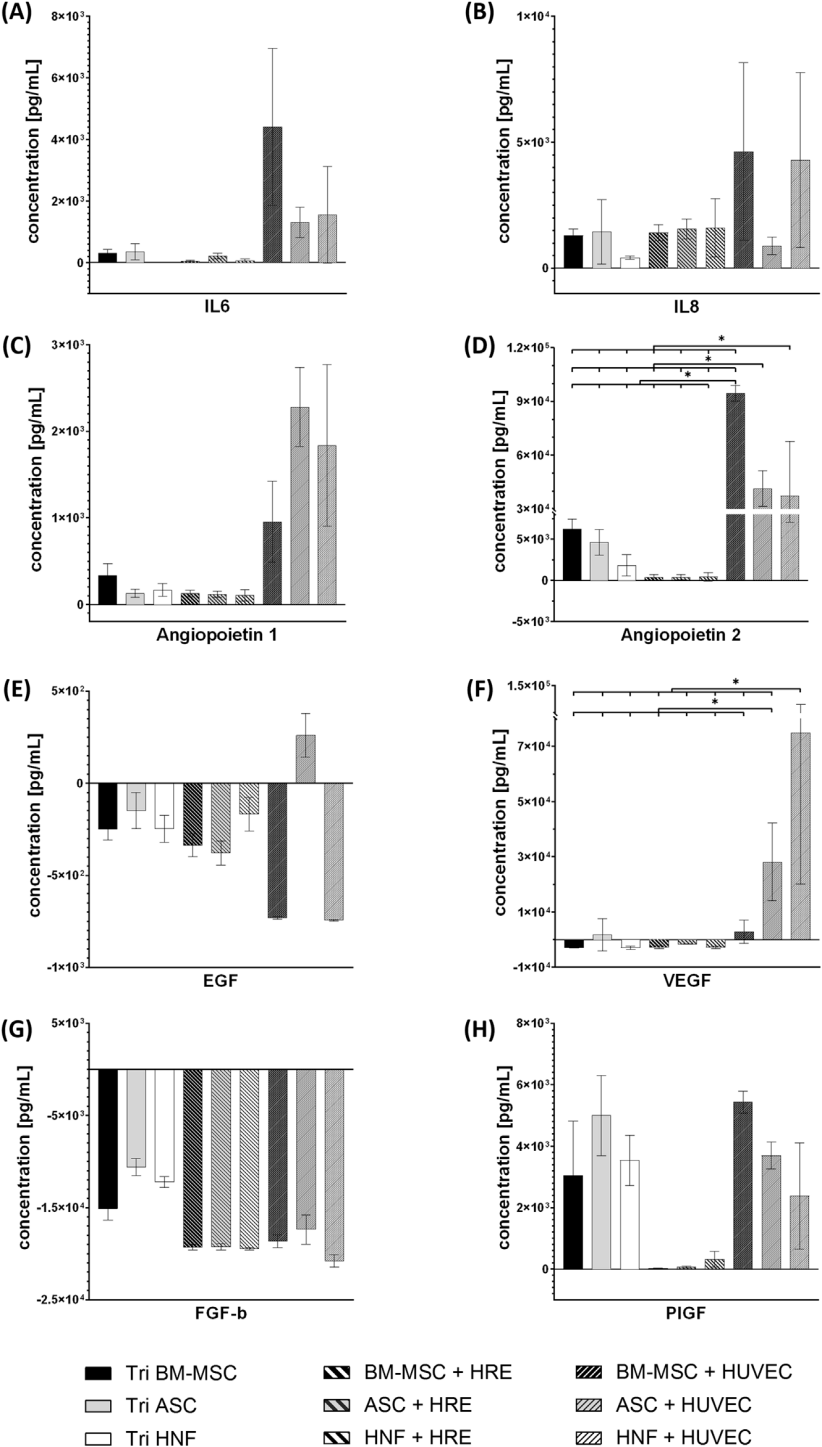
LEGENDPlex immunoassay for cytokines associated with angiogenesis and inflammation. Concentrations of cytokines IL6 **(A)**, IL8 **(B)**, angiopoietin 1 **(C)**, angiopoietin 2 **(D)**, EGF **(E)**, VEGF **(F)**, FGF-b **(G)** and PIGF **(H)** in tri-cultures, co-cultures with HRE and co-cultures with HUVEC using different supporting cell types. Concentrations have been normalized to cell-free medium. Error bars show standard deviation. Statistical significance indicated by “*” shows significance (*p* < 0.05) between mean values.

In order to investigate differences in cytokine production between the cell types, media supernatants were analyzed for levels of eight cytokines involved in vascularization processes and inflammation by LEGENDPlex bead-based immunoassay (Figure 8). Cytokine concentrations were normalized to cell-free media. Statistically significant differences could be observed for angiopoietin 2 (Figure 8D) and VEGF (Figure 8F). The HUVEC-co-culture with BM-MSC showed significantly increased levels of angiopoietin 2 (94,513.38 ± 4,400.76 pg/ml) in comparison to all other groups. HUVEC-co-cultures with ASC (41,359.83 ± 9,893.24 pg/ml) and HNF (37,400.70 ± 30,372.09 pg/ml) each also revealed to have significantly increased levels in angiopoietin 2 in comparison to tri-cultures and co-cultures with HRE (Figure 8D).

HUVEC-co-cultures with ASC or HNF in addition exhibited significantly higher levels of VEGF (28,160.08 ± 14,049.48 pg/ml resp. 74,956.75 ± 54,838.28 pg/ml) when compared to all other groups (Figure 8F). Differences in cytokine levels of IL6, IL8, angiopoietin 1, EGF, FGF-b and PIGF (Figures 8A–C,E, G-H) were found to be statistically non-significant. Nevertheless, trends can be observed: HUVEC-co-cultures with BM-MSC seemed to have increased levels in IL6, IL8 and angiopoietin 1 when compared to the other groups. The latter was also found to be high in the two other HUVEC-co-cultures whereas only the HUVEC-co-culture with ASC seemed to have increased IL8 levels. IL6 concentrations in HRE-co-cultures with ASC or HNF and in tri-cultures with HNF were close to the detection limit (Figures 8A–C). With exception of the HUVEC-co-culture with ASC, all cultures consumed EGF (Figure 8E) while FGF-b was consumed by all cultures without exception (Figure 8G). High levels of PIGF were found for all tri-cultures and co-cultures with HUVEC while concentrations in HRE-co-cultures with MSC were close to the detection limit (Figure 8H). However, these observations have to be described as numerical differences without statistical significance demonstrated in this study. Cytokine levels in media supernatants of monocultures with BM-MSC, ASC or HNF in fibrin gel are given in the supplementary (see Supplementary Figure S7). IL6 concentration was found to be significantly higher in BM-MSC-monocultures while angiopoietin 1 level was significantly increased in monocultures of HNF. In addition, concentrations of pro-inflammatory cytokines IL33, TSLP and TGF-β1 in media supernatants are given in the supplementary (see Supplementary Figure S8). While levels of IL33 and TSLP were found to be very low in all cultures, TGF-β1 could be detected to some degree in co-cultures with HUVEC as well as in monocultures of supporting cell types.

In summary, using BM-MSC as supporting cell type in tri-cultures led to well differentiated respiratory epithelium and a high degree of vascularization. HNF-supported tri-cultures also showed mucociliary differentiation and formation of vascular-like structures although to a lesser degree than with the BM-MSC. HRE in tri-cultures containing ASC were mostly undifferentiated while differences in vascularization were marginal. Co- and tri-cultures containing BM-MSC or HNF contained a basal cell layer positive for pan-cytokeratin, ciliated cells, mucus-producing goblet cells, tight junctions and epithelial cytoskeleton comparable to native tissue. Although ciliation amount in HNF-tri-cultures was comparable to tri-cultures with BM-MSC, and HNF-tri-cultures were also found to be positive for epithelial cell marker expression, epithelial morphology observed in PAS reaction and immunohistochemistry more closely resembled the native state in BM-MSC-tri-cultures with regard to cell arrangement, cell marker localization and layer thickness. Moreover, vascularization in tri-cultures with BM-MSC associated with a significantly higher number of branching points compared to all other cultures. Although differences in area and length of the vascular-like structures were only found to be statistically significant for BM-MSC-tri-cultures in comparison to co-cultures with ASC or HNF, an overall trend towards higher values in BM-MSC-tri-cultures was observed.

## Discussion

To date, strategies for tracheal tissue engineering hardly reach translation into the clinic as they face three major challenges: poor mechanical properties of used scaffolds, inadequate vascularization and insufficient epithelialization (Zhang et al., 2015; Soriano et al., 2021; Sun et al., 2021). Nevertheless, *in vitro* culture of respiratory epithelial cells has become a common method in airway disease research nowadays. ALI-cultures have proven to be a suitable platform for studying viral infection pathways, drug testing, as well as toxicological assays and are widely used to model common respiratory diseases (Cao et al., 2021). However, it has become apparent that more than 1 cell type is needed to create a functional airway mucosa (Zhang et al., 2015). Most studies that combine respiratory epithelium with other cell types have focused on fibroblasts or feeder cells (Albers et al., 2015; Law et al., 2016; Hiemstra et al., 2019; Lodes et al., 2020; Bukowy-Bieryllo, 2021), whereas only few reports on co-culture systems with MSC or endothelial cells exist (Le Visage et al., 2004; Franzdottir et al., 2010; Law et al., 2016; Blume et al., 2017; Taniguchi et al., 2018). To our knowledge, our hydrogel-based tri-culture system first published in 2019 combining primary respiratory epithelium, primary endothelial cells and primary supporting cell types in direct contact to mimic the respiratory mucosa is unique in literature (Kreimendahl et al., 2019).

In this study, influence of different supporting cell types, BM-MSC, ASC and HNF, on *in vitro* differentiation of HRE and formation of vascular-like structures mediated by HUVEC is analyzed. Because HNF had already proven to promote mucociliary differentiation and vascularization in tri-cultures with HUVEC and HNEC (Kreimendahl et al., 2019), they were used as controls. In addition, we examined epithelial differentiation and formation of vascular-like structures in co-cultures of supporting cell types with HRE or HUVEC.

We found BM-MSC to be superior to ASC and HNF with regard to mucociliary differentiation and vascularization both in tri-cultures and in co-cultures. This was followed by tri-cultures with HNF while tri-cultures containing ASC did not promote mucociliary differentiation. These results stand in some contrast to the results obtained in our previous study where we established the tri-culture system based on HNF, HUVEC and HNEC. Morphology of HNF-tri-cultures with HNEC was found to be more similar to the BM-MSC-tri-cultures presented in this study. The different origin of the epithelial cells can explain those differences. There is evidence in the literature that gene expression differs between airway epithelial cells from nasal and bronchial origin and that these cell types cannot be expected to behave in the same way (Imkamp et al., 2019).

### Mucociliary Differentiation

Interestingly, tri-culture reduced ciliation in comparison to the corresponding co-culture. The presence of a third cell type in the tri-culture system resulting in higher consumption of nutrients and growth factors might be a possible reason. Increasing the media exchange frequency or extension of the ALI period could therefore be strategies to increase ciliation in tri-cultures. However, this was not tested in this study in order to keep all parameters constant. In contrast, this effect was not generally discovered for vascularization. Based on the mucociliary differentiation observed in the co-culture of HUVEC and HRE (see Supplementary Figure S1 and Supplementary Figure S5), a negative influence of HUVEC on the ciliation is not indicated. Positive α-tubulin staining inside BM-MSC found in BM-MSC-tri- and co-cultures can be explained by detection of intracellular microtubuli as part of the cytoskeleton, since α-tubulin acts as marker for microtubuli not only in cilia (Zhou et al., 2017).

Most studies dealing with airway epithelial co-cultures use fibroblasts as supporting cell type (Hiemstra et al., 2019). Their ability to promote epithelial cell differentiation has been confirmed in several studies (Albers et al., 2015; Abs et al., 2019). In our first study that established the tri-culture system based on HNEC, we therefore used HNF as supporting cell type. Nevertheless, access to fibroblasts from respiratory origin might be limited for tissue engineering applications as it requires painful surgery and can be associated with high donor variability depending on the patient´s medical history (Law et al., 2020; Luengen et al., 2020). MSC provide high potential for autologous cell use as they can be obtained in high numbers through rather minimal invasive procedures (Mushahary et al., 2018). Furthermore, Taşkiran *et al.*, who compared differences in molecular signatures of BM-MSC and dermal fibroblasts, recommend the use of BM-MSC for developmental and differentiation investigations (Taşkiran and Karaosmanoğlu, 2019). Although MSC and fibroblasts share features in tissue remodeling, angiogenesis and wound healing, fibroblasts bear the risk of hyperproliferation and fibrosis while MSC are known to have anti-fibrotic properties (Zupan, 2021). Taken together, these investigations support our findings regarding the ability of BM-MSC to promote *in vitro* differentiation of respiratory epithelial cells. Although co-cultures with ASC exhibited a low degree of cilia and mucus formation, the corresponding tri-cultures lacked signs of complete mucociliary differentiation. This correlated with the general reduction of mucociliary differentiation in tri-cultures when compared to co-cultures. However, epithelial cell layers in ASC-co-cultures were unusually thick and deficient in cell polarization and a basal cell layer, showing signs of epithelial hyperproliferation. These results go in line with observations made by Kobayashi *et al.*, who found mucin5AC and β-tubulin expression of tracheal epithelial cells *in vitro* to be reduced in co-cultures with ASC compared to co-cultures with fibroblasts. Moreover, the authors could show an enhanced epithelial proliferation in ASC-co-cultures (Kobayashi et al., 2010).

### Vascularization

With respect to vascularization, both MSC and fibroblasts are known to have pro-angiogenic properties (Zupan, 2021). Several investigations could prove the ability of both cell types to promote formation of vascular-like structures *in vitro* (Rao et al., 2012; Freiman et al., 2016; Lee et al., 2016). In a previous study published by our group, we found both fibroblasts and MSC of different origins to be suitable as supporting cell types for vascularization *in vitro*, with BM-MSC showing a widespread network of smaller vascular-like structures and more branching points in co-culture with HUVEC compared to the other cell types (Kniebs et al., 2020). This matches our results, although the effect was only visible in BM-MSC-tri-cultures and not as strong in co-cultures. Lumen formation observed for all tri-cultures (see Supplementary Figure S6) indicates presence of essential ECM components known to be synthesized by supporting cells (Newman et al., 2011).

### Cytokine Production

In this study, we analyzed levels of cytokines IL6, IL8, angiopoietin 1, angiopoietin 2, EGF, VEGF, FGF-b and PIGF known to be involved in vascularization (Lovett et al., 2009; Amirsadeghi et al., 2020; Sun et al., 2021). Rather high standard deviations observed in LEGENDPlex assays result from donor-dependent variation. Donor tissue was derived from patients with unknown medical history and was treated anonymously due to reasons of data protection. Therefore, it cannot be precluded that a patient´s medical history may influence the cell behavior *in vitro*. In general, primary cell differentiation is often connected to high donor-to-donor variability (Russell et al., 2018). Statistically significant differences in the LEGENDPlex assays were only found for VEGF and angiopoietin 2. VEGF is known to promote vasculogenesis by increasing cell proliferation, vascular permeability and vasodilatation (Melincovici et al., 2018). In addition, its expression was documented in a broad variety of tissues and cell types including respiratory epithelial cells (Melincovici et al., 2018; Luengen et al., 2020). As VEGF is supplemented in endothelial cell growth medium 2 (EGM2) with a concentration of 0.5 ng/ml and might additionally be present in the fetal calf serum (FCS) supplement as well, most cultures were found to consume VEGF, while HUVEC-co-cultures with ASC and HNF showed strong VEGF production. This might be a sign for still ongoing vascularization processes in these two cultures. In monocultures of BM-MSC, ASC and HNF, VEGF and angiopoietin 2 production was comparably low (see Supplementary Figure S7). The role of angiopoietin 2 in vascularization and angiogenesis is understood to be highly regulated and context-dependent as it can act as either agonist or antagonist to the potent pro-angiogenic growth factor angiopoietin 1 by binding to the same Tie2 receptor (Akwii et al., 2019). Among other parameters, increased VEGF concentration contributes to the pro-angiogenic activity of angiopoietin 2 (Akwii et al., 2019). Interestingly, we observed VEGF consumption and little angiopoietin 2 production in co-cultures with HRE. These findings support the concept of respiratory epithelial cells and endothelial cells promoting each other in differentiation (Eichmann and Simons, 2012; Luengen et al., 2020). Our data suggest that HRE additionally require VEGF for differentiation and that differentiating HRE also trigger formation of vascular structures. Combining the vascularization and differentiation outcomes with VEGF and angiopoietin 2 levels, it appears that supporting cells assist in providing the necessary growth factor levels, especially in the context of high VEGF-demand by both HUVEC and HRE.

Regarding growth factors IL6, IL8, angiopoietin 1, EGF, FGF-b and PIGF, differences between cultures were found to be non-significant. The observed trend towards higher PIGF levels in cultures containing HUVEC can be explained by the fetal origin of the cells. The protein belonging to the VEGF family was found to be involved in several physiological and pathological vascularization processes. Although its pro-angiogenic effects might be beneficial for vessel formation, increased PIGF levels are connected to pathological angiogenic processes (Shibuya, 2008). In order to avoid potential undesirable effects of PIGF and in addition enhance availability of autologous endothelial cells, HUVEC could be replaced in future experiments by adipose-derived microvascular endothelial cells (Freiman et al., 2018). As angiopoietin 1 is well-characterized with regard to its strong angiogenic effects, higher levels in co-cultures with HUVEC in comparison to tri-cultures support the assumption of upregulated vascularization processes in these cultures (Akwii et al., 2019). Interestingly, a significantly higher production of angiopoietin 1 in the HNF monoculture was not reflected in tri- and co-cultures revealing the importance of cell type interactions for changes in cytokine expression (see Supplementary Figure S7). Next to promoting vascularization, EGF is also known to be involved in mucociliary differentiation of respiratory epithelial cells (Cozens et al., 2018; Luengen et al., 2020). The same applies for FGF-b, which is known to participate in both epithelial repair and angiogenesis processes (Murakami and Simons, 2008; Guzy et al., 2015). Both IL6 and IL8 are pro-inflammatory cytokines, and although inflammation represents a necessary component in tissue regeneration and vascularization, de-regulation and high levels of both can contribute to chronic inflammation and cancer development (Eming et al., 2017; Kaur et al., 2020; Fousek et al., 2021). Altogether, the low interleukin levels observed in the tri-cultures are promising with regard to future applications of the mucosa model. However, the generally available data on cytokines required for successful mucociliary differentiation of HRE is insufficient beyond the cytokines VEGF, EGF and FGF-b. While well-defined and established cytokine panels for vascularization analysis exist, only little is known on the intrinsic factors that finally lead to a mucociliary phenotype. On the other hand, several pro-inflammatory factors are involved in pathological processes of the diseased airway epithelium: IL33, TSLP and TGF-β1 are known regulators in common airway diseases like asthma, allergic rhinitis, COPD or cystic fibrosis (Aschner and Downey, 2016; Stewart et al., 2018; Hong et al., 2020). The low levels of the epithelial “alarmins” IL33 and TSLP (Mitchell and O'Byrne, 2017), that have been found in our study (see Supplementary Figure S8) contribute to the promising results already observed with low IL6 and IL8 levels. And although TGF-β1 was detected in co-cultures with HUVEC and monocultures of supporting cell types (see Supplementary Figure S8), levels in HRE cultures were observed to be very low. Taken together, these results may in future qualify our model to be used as *in vitro* platform for studies on airway diseases.

## Conclusion

Further research is needed to evaluate the perfusability of the lumen-forming vascular-like structures and the options to connect the vascular network to the patient’s blood vessels during implantation. Furthermore, creation of upscaled mucosal patches and the combination with a suitable cartilage-like scaffold would represent the next steps towards the development of an implantable tissue-engineered tracheal replacement. In addition, employment of autologous respiratory epithelial cells derived from diseased patients could provide valuable information for new therapeutic approaches. Such further development towards an *in vitro* disease model might replace animal experiments in research on respiratory diseases like asthma or COVID-19.

In this study, we compared the influence of BM-MSC, ASC and HNF on the mucociliary differentiation of HRE and on the formation of vascular-like structures mediated by HUVEC in a fibrin-based *in vitro* tri-culture model. We were able to reproducibly demonstrate that BM-MSC promote both epithelial differentiation and vascularization most effectively, followed by HNF. In summary, analysis of growth factor content in media supernatant showed reduced levels of pro-inflammatory and angiogenic cytokines in tri-cultures and HRE-co-cultures in comparison to co-cultures with HUVEC. Thus, our BM-MSC-based model of the respiratory mucosa providing a pseudostratified ciliated epithelium including mucus-producing goblet cells and a vasculature network marks a major step on the way to a tissue-engineered tracheal implant.

## Data Availability

The datasets generated and analyzed during the current study are available from the corresponding author on reasonable request.

## Glossary

ABM: antibiotic/antimycotic
AECGM: airway epithelial cell growth medium
ALI: air-liquid interface
ANOVA: analysis of variance
ASC: adipose-derived mesenchymal stromal cells
BM-MSC: bone marrow-derived mesenchymal stromal cells
BPE: bovine pituitary extract
DMEM: Dulbecco’s Modified Eagle Medium
DMSO: Dimethyl sulfoxide
ECM: extracellular matrix
EGF: epidermal growth factor
EGM2: endothelial cell growth medium 2
EMM: EGM2-MucilAir-Mixture
FCS: fetal calf serum
FGF-b: fibroblast growth factor basic
HNEC: human nasal epithelial cells
HNF: human nasal fibroblasts
HRE: human respiratory epithelial cells
HUVEC: human umbilical vein endothelial cells
IL33: interleukin-33
IL6: interleukin-6
IL8: interleukin-8
KSFM: keratinocyte-SF medium
mAir: modified airway epithelial cell growth medium
MSC: mesenchymal stromal cells
NGS: normal goat serum
PAS reaction: periodic acid-Schiff’s reaction
PIGF: placenta growth factor
SEM: scanning electron microscopy
TGF-β1: transforming growth factor-beta1
TPLSM: two-photon laser scanning microscopy
TSLP: thymic stromal lymphopoietin
VEGF: vascular endothelial growth factor
WHO: World Health Organization
