# Supplementary material for: Bone Marrow Derived Mesenchymal Stromal Cells Promote Vascularization and Ciliation in Airway Mucosa Tri-Culture Models in Vitro
Source: Front Bioeng Biotechnol. 2022 Jun 17;10:872275. doi: 10.3389/fbioe.2022.872275 (PMC9247357; doi:10.3389/fbioe.2022.872275)
Supplement: Supplementary file 1 [file DataSheet1.docx]

Supplementary Material

# **1 Supplementary Data**

#

**1.1 Supplementary Figures**


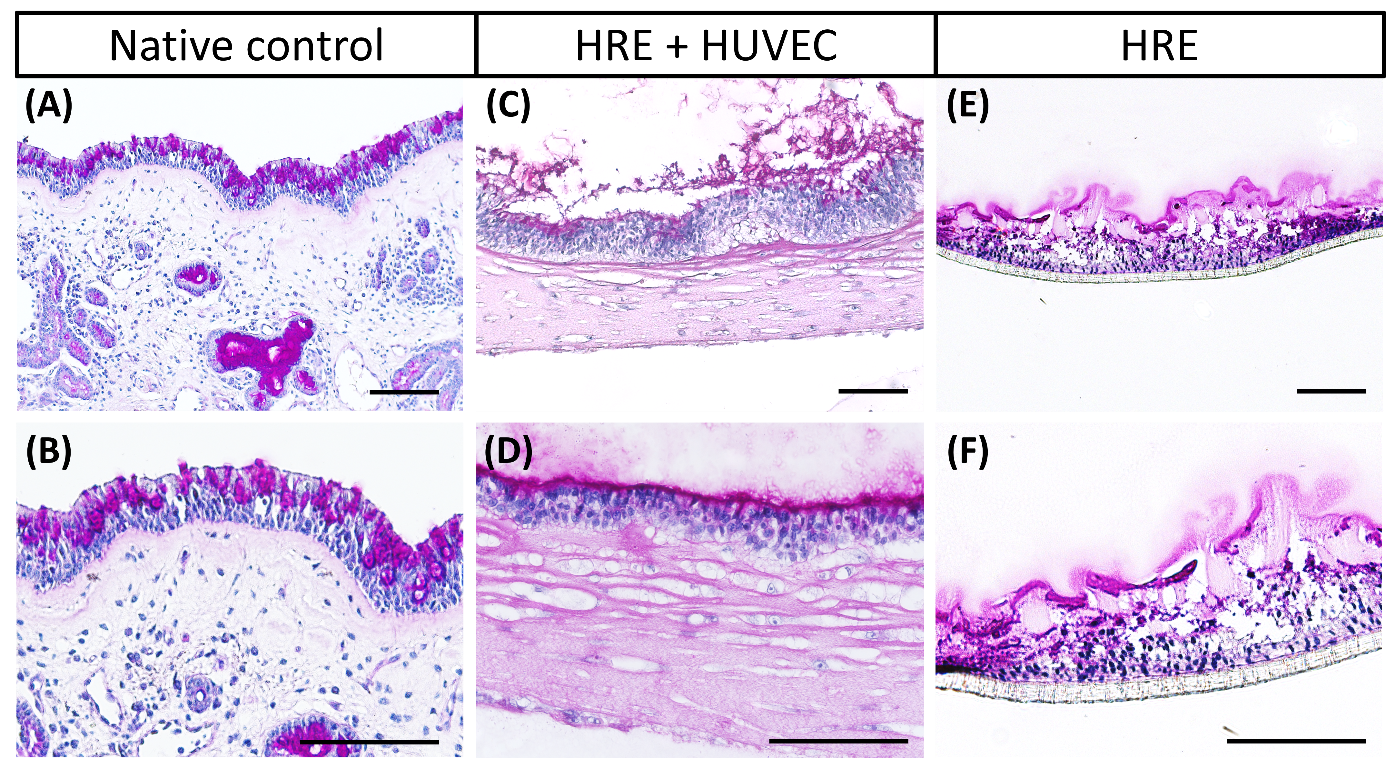


**Supplementary Figure S1: PAS reaction of native tissue, a co-culture of HRE and HUVEC and a HRE monoculture. (A, B)**: PAS reaction revealed presence of glycogens, mucopolysaccharides and glycoproteins in native human nasal concha tissue; **(C, D)**: PAS reaction showed overall morphology of fibrin gels containing HUVEC and HRE seeded on top; **(E, F)**: PAS reaction of HRE in monoculture. Representative pictures of two different magnifications are shown. Scale bar: 100 µm.


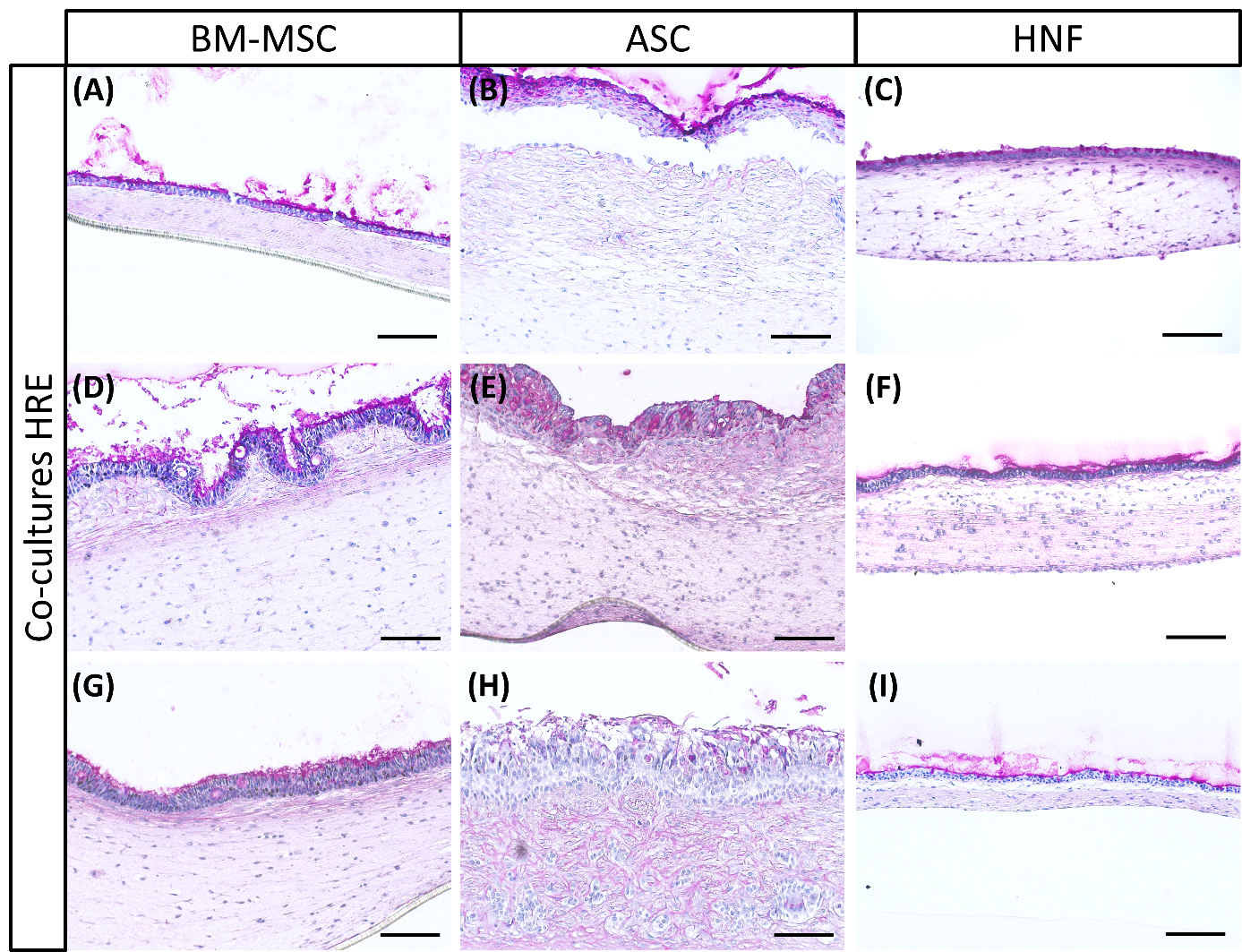


**Supplementary Figure S2: PAS reaction of all co-cultures of HRE with supporting cell types BM-MSC, ASC or HNF. (A, D, G)**: PAS reaction of HRE-co-cultures with three BM‑MSC donors; **(B, E, H)**: PAS reaction of three ASC-donors in co-culture with HRE; **(C, F, I)**: co-cultures of three HNF-donors with HRE stained by PAS reaction. Scale bar: 100 µm.


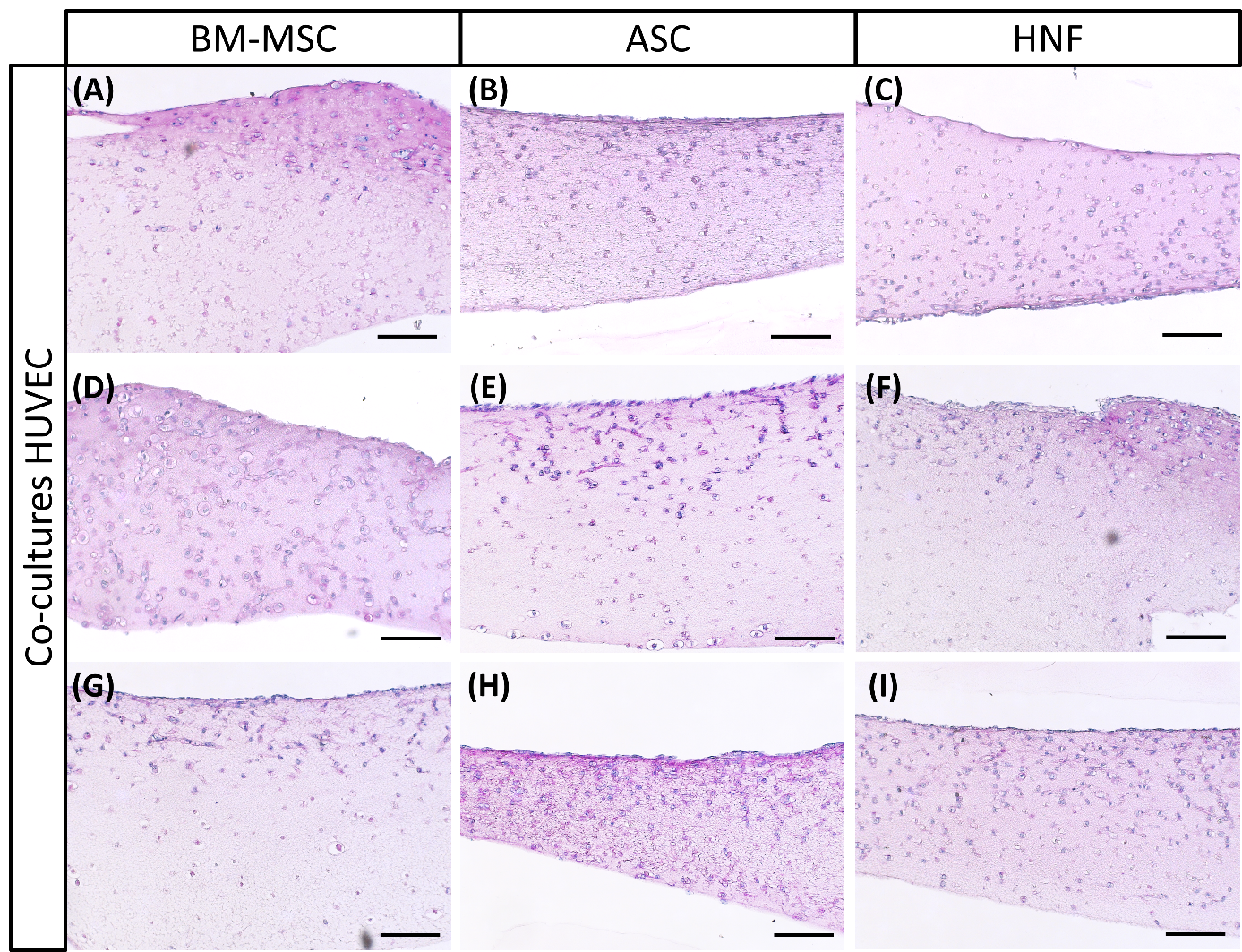


**Supplementary Figure S3: PAS reaction of all co-cultures of HUVEC with supporting cell types BM-MSC, ASC or HNF. (A, D, G)**: PAS reaction of HUVEC-co-cultures with three BM‑MSC donors; **(B, E, H)**: PAS reaction of three ASC-donors in co-culture with HUVEC; **(C, F, I)**: co-cultures of three HNF-donors with HUVEC stained by PAS reaction. Scale bar: 100 µm.


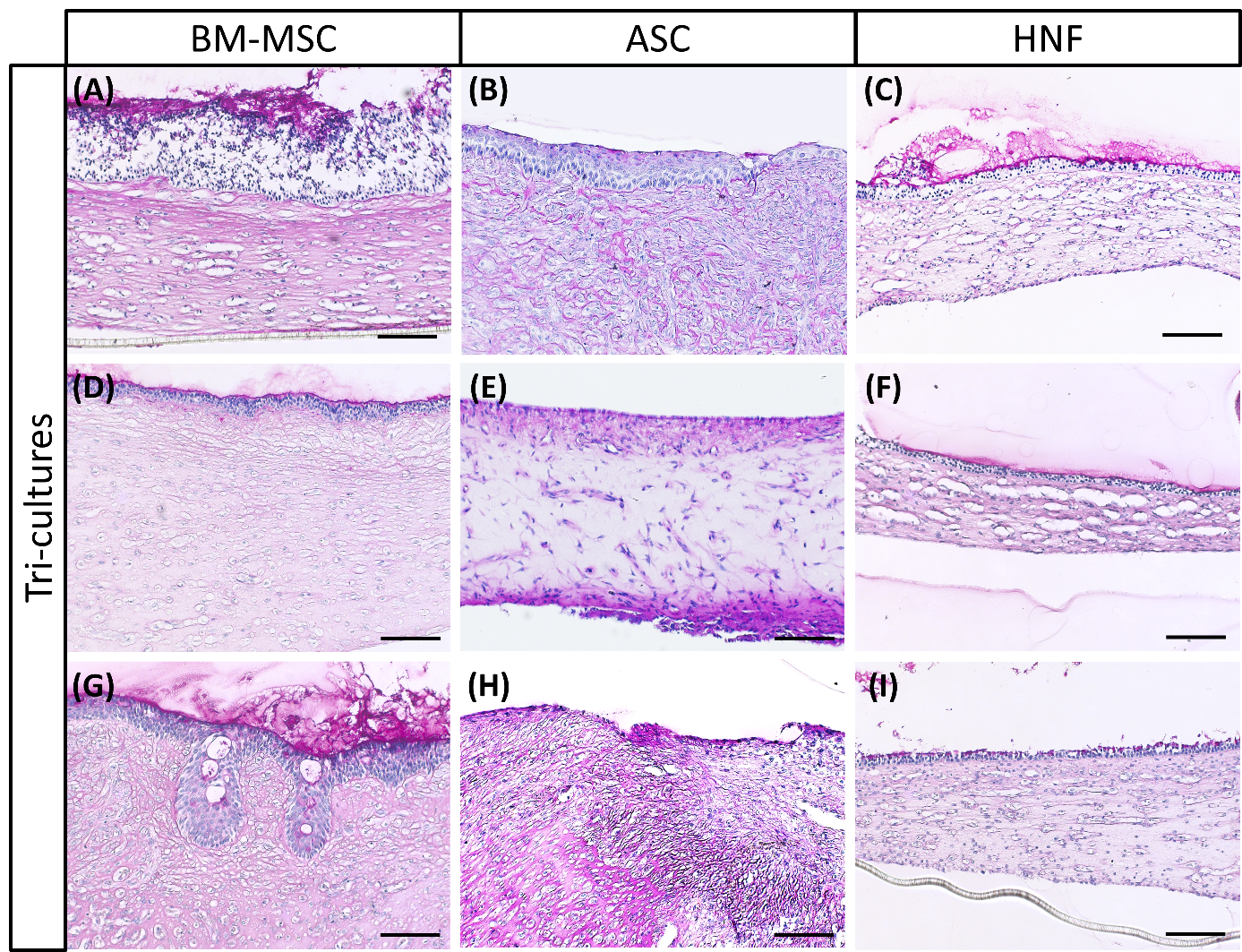


**Supplementary Figure S4: PAS reaction of all tri-cultures with supporting cell types BM-MSC, ASC or HNF. (A, D, G)**: PAS reaction of tri-cultures with three BM‑MSC donors; **(B, E, H)**: PAS reaction of three ASC-donors in tri-culture with HUVEC and HRE; **(C, F, I)**: tri-cultures of three HNF-donors with HUVEC stained by PAS reaction. Scale bar: 100 µm.


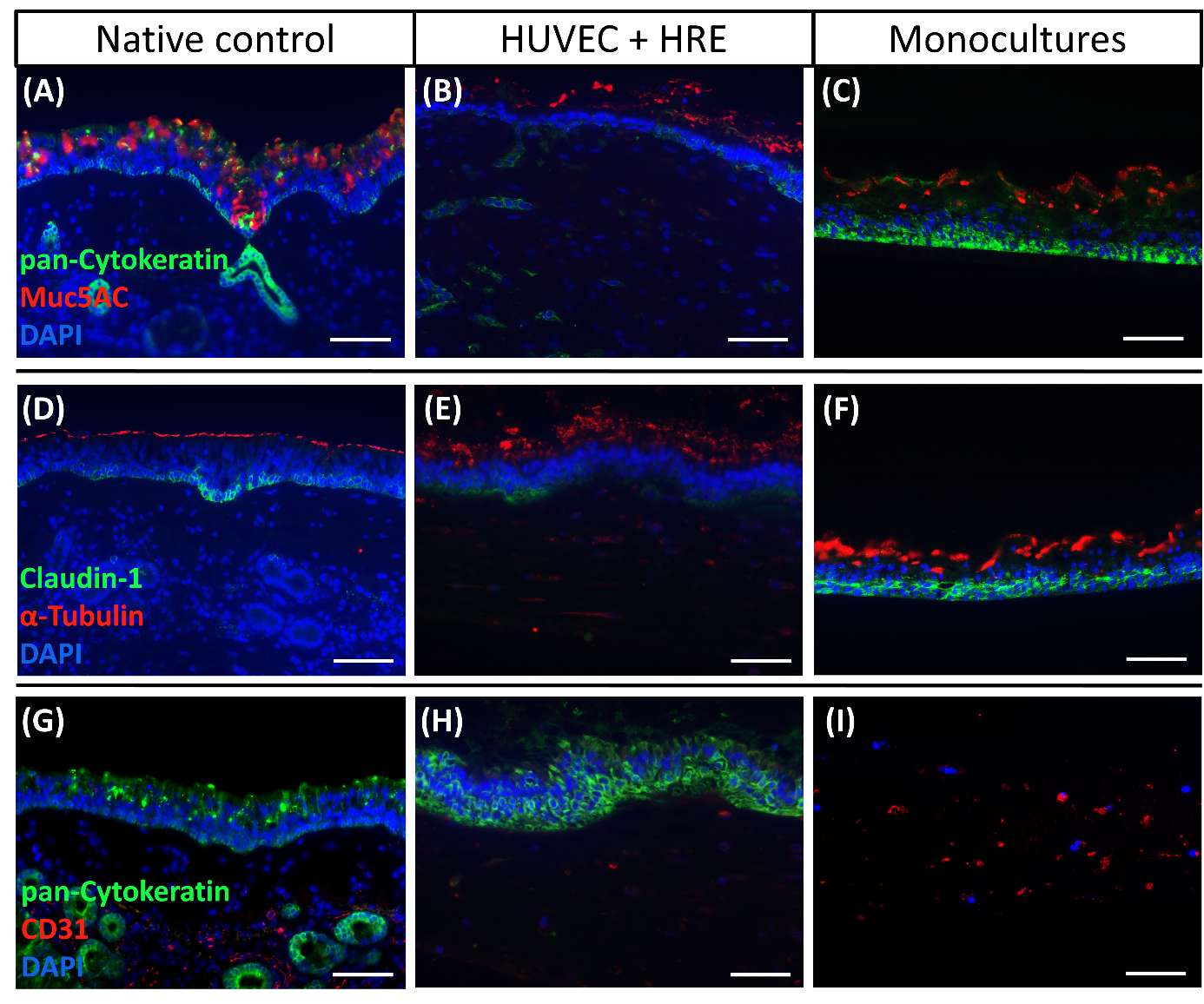


**Supplementary Figure S5: Immunohistochemical staining of native tissue, a co-culture of HRE and HUVEC and monocultures of HRE and HUVEC. (A-C)**: pan-cytokeratin (green) was used to detect epithelial keratins while mucin5AC (red) showed presence of mucus and goblet cells in native human nasal concha tissue **(A)**, in the HUVEC-HRE-co-culture **(B)** and in the HRE-monoculture **(C)**; **(D-F)**: claudin-1 (green) visualized tight junction formation and α-tubulin (red) stained cilia in native human nasal concha tissue **(D)**, in the HUVEC-HRE-co-culture **(E)** and in the HRE-monoculture **(F)**; **(G, H)**: pan-cytokeratin stained the epithelial cell layer while CD31 was used to detect endothelial cells in native human nasal concha tissue **(G)** and in the HUVEC-HRE-co-culture **(H)**; **(I)**: CD31 stainings of HUVEC in monoculture; DAPI (blue) was used to counterstain cell nuclei. Representative pictures are shown. Scale bar: 50 µm.


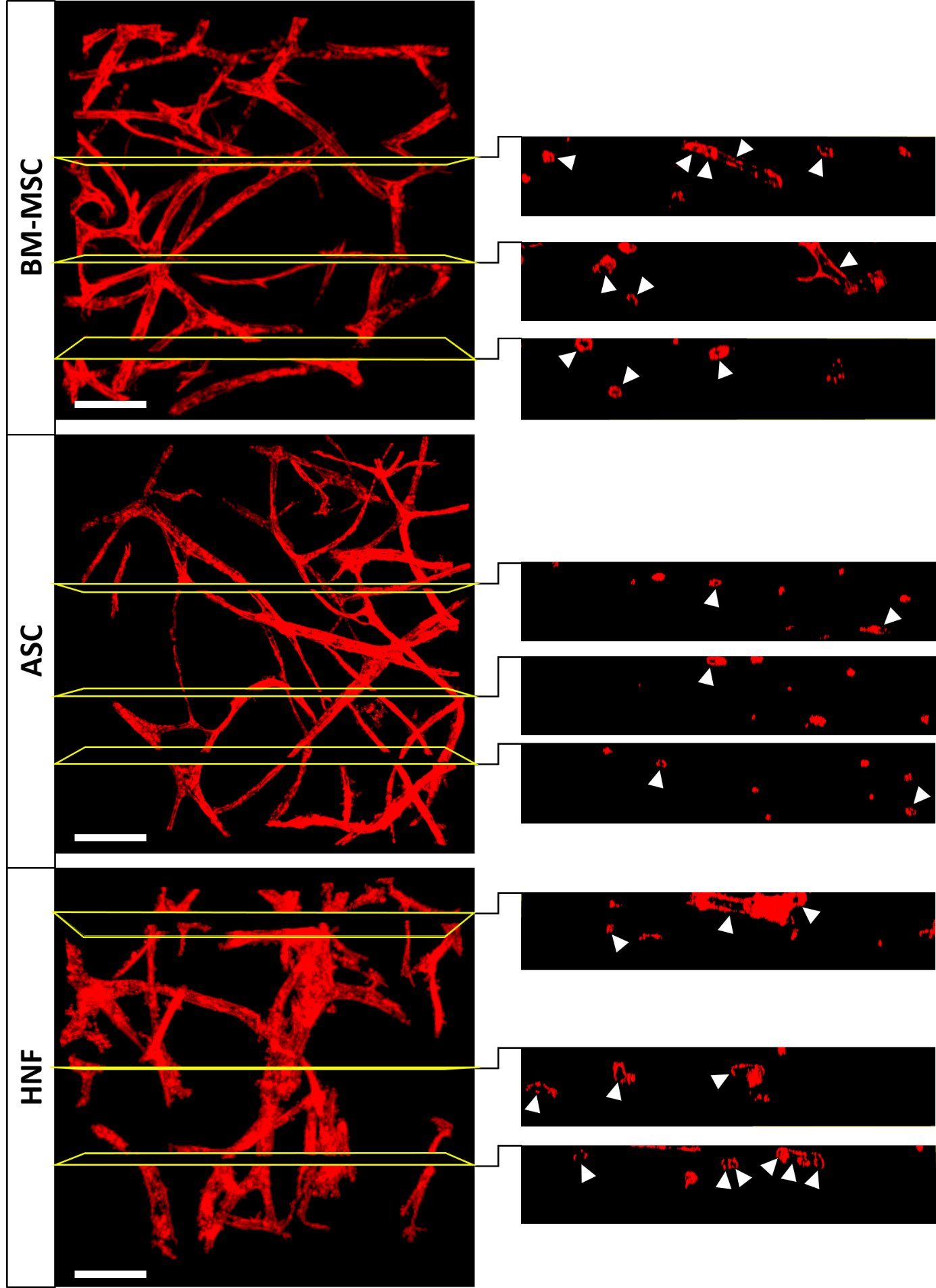


**Supplementary Figure S6: Lumen formation in tri-cultures with BM-MSC, ASC and HNF based on cross sections of TPLSM images.** CD31-staining visualized vascular-like structures in the fibrin gels, lumen in the cross sections are indicated by white triangles. Scale bar: 50 µm.

**Supplementary Figure S7:** **LEGENDPlex immunoassay for angiogenic cytokines in monocultures of BM-MSC, ASC or HNF.**  Concentrations of cytokines IL6, angiopoietin 1, angiopoietin 2, EGF, FGF-b, IL8, PIGF and VEGF in media supernatants of monocultures using different supporting cell types. Concentrations have been normalized to cell-free medium. Error bars show standard deviation. Statistical significance indicated by “*” shows significance (p < 0.05) between mean values.


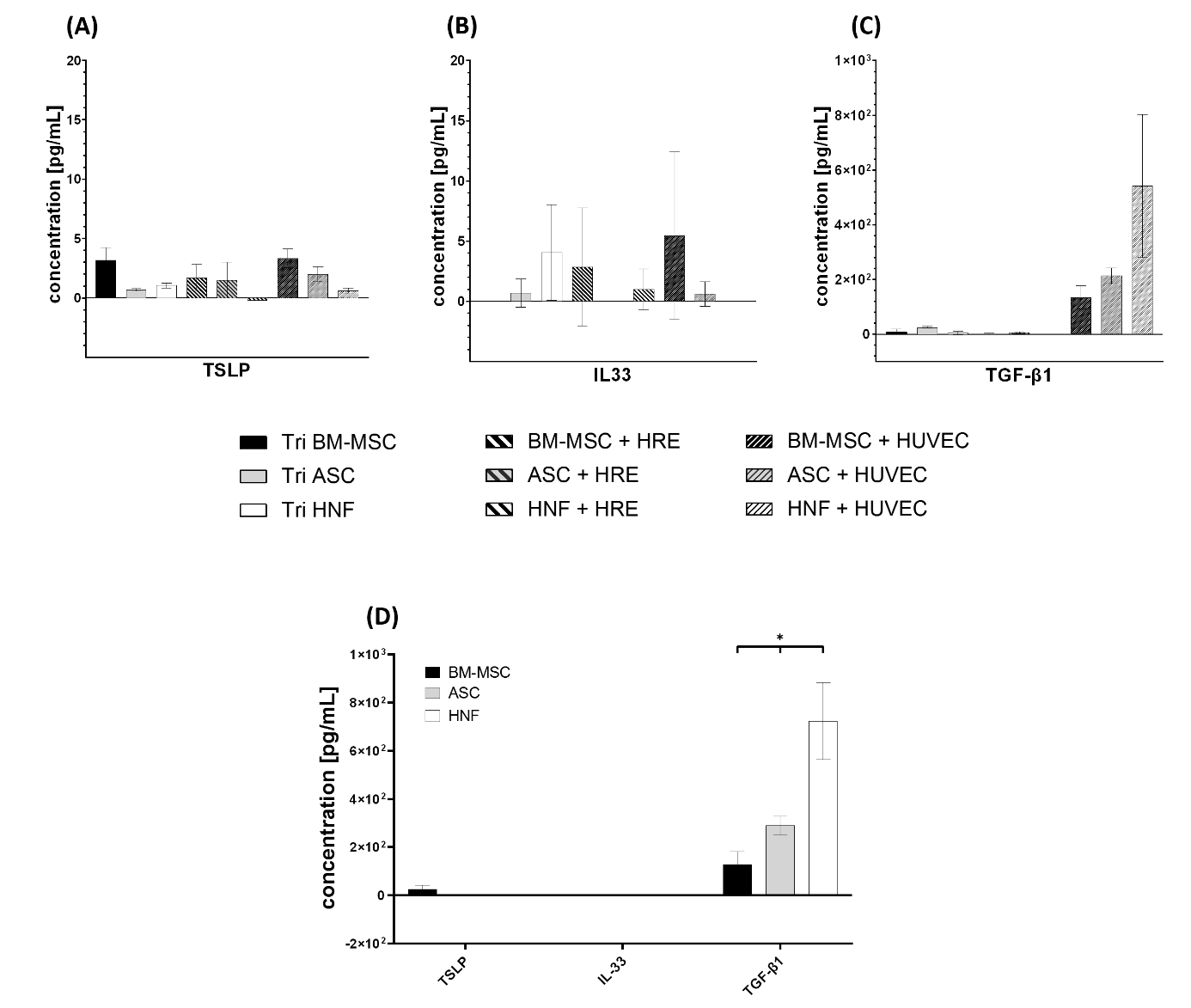


**Supplementary Figure S8: LEGENDPlex immunoassay for cytokines TSLP, IL33 and TGF-β1 in tri-cultures, co-cultures with HRE, co-cultures with HUVEC and in monocultures of BM-MSC, ASC or HNF.**  Concentrations of cytokines IL33 (**A**), TSLP (**B**) and TGF-β1 (**C**) in media supernatants of tri-cultures and all co-cultures as well as monocultures (**D**) using different supporting cell types. Concentrations have been normalized to cell-free medium. Error bars show standard deviation. Statistical significance indicated by “*” shows significance (p < 0.05) between mean values.
